# Malnutrition in Elderly Patients with Chronic Kidney Disease—The Role of Albuminuria

**DOI:** 10.3390/life15060898

**Published:** 2025-05-31

**Authors:** Diana Moldovan, Ina Kacso, Lucreția Avram, Cosmina Bondor, Crina Rusu, Alina Potra, Dacian Tirinescu, Maria Ticala, Ariana Condor, Dana Crisan, Valer Donca

**Affiliations:** 1 Department of Nephrology, Faculty of Medicine, “Iuliu Hatieganu” University of Medicine and Pharmacy Cluj-Napoca, 400012 Cluj-Napoca, Romania; maria.kacso@umfcluj.ro (I.K.); claudia.rusu@umfcluj.ro (C.R.); alina.potra@umfcluj.ro (A.P.); cosa.maria@umfcluj.ro (M.T.); 2 Department of Geriatrics—Gerontology, Faculty of Medicine, “Iuliu Hatieganu” University of Medicine and Pharmacy Cluj-Napoca, 400012 Cluj-Napoca, Romania; valer.donca@umfcluj.ro; 3 Clinical Municipal Hospital, 400139 Cluj-Napoca, Romania; 4 Department of Medical Informatics and Biostatistics, “Iuliu Hatieganu” University of Medicine and Pharmacy Cluj-Napoca, 6 Pasteur Street, 400349 Cluj-Napoca, Romania; cbondor@umfcluj.ro; 5 Department of Internal Medicine, 5th Medical Clinic, Faculty of Medicine, “Iuliu Hatieganu” University of Medicine and Pharmacy Cluj-Napoca, 400012 Cluj-Napoca, Romania

**Keywords:** malnutrition, Mini Nutritional Assessment, geriatric nutritional risk index, bioelectrical impedance analysis, chronic kidney disease, albuminuria, elderly

## Abstract

**Background**: The global population is rapidly aging, and an epidemic increase in chronic kidney disease (CKD) has been reported. As the presence of malnutrition in elderly CKD patients can pose serious health problems, the aim of our study was to identify, using different assessment tools, the relationship between nutrition with kidney function and albuminuria in elderly patients. **Methods**: The study included 793 hospitalized patients aged 65 years and older. A comprehensive assessment of nutritional status and renal involvement was performed, and the relationship between malnutrition and kidney issues was tested. **Results**: CKD was highly prevalent in our geriatric population, with 39.84% having CKD G3a–5. Malnutrition, determined according to the Mini Nutritional Assessment (MNA) score, was identified in 34.6% of patients. With an increase in albuminuria, we observed worse nutrition indicators: low serum albumin; lower body fat (*p* = 0.002) and visceral fat (*p* = 0.001), assessed via bioimpedance; and lower MNA (*p* = 0.04) and geriatric nutritional risk index (GNRI) (*p* = 0.002) scores. Elderly patients with CKD G3a–5 had lower HDL-cholesterol (*p* < 0.001), higher triglycerides (*p* < 0.001), lower albumin (*p* = 0.011), and a lower MNA score (*p* = 0.001). **Conclusions**: Malnutrition was found to be common and more severe with increased albuminuria and decreased eGFR. Our study sheds light on a novel relationship between malnutrition, albuminuria, and renal function in a geriatric population.

## 1. Introduction

Living in a society characterized by an aging population is one of humanity’s main concerns about the future. Aging is characterized by diminishing organ reserves; weakened homeostatic body controls; and a loss of functionality, autonomy, and social roles. In 2024, around 10% of people worldwide were older than 65 years, whereas this age group is expected to account for >16% of the global population by 2050 [1]. Europe is expected to experience one of the greatest aging rates, with 28.9% of its populations aged > 65 years in 2050. Notably, the increasing number of older adults occurs in the context of record-low birth rates in many populations, especially those in Eastern European countries [1]. Population aging is associated with an increased incidence and duration of non-communicable diseases, including chronic kidney disease (CKD), leading to a serious burden on healthcare systems.

Diagnosing CKD in older individuals presents specific challenges. The KDIGO criteria diagnose CKD as an estimated glomerular filtration rate (eGFR) of <60 mL/min/1.73 m^2^ or urinary abnormalities, such as albuminuria, that are persistent for more than 3 months, regardless of age [2]. While age-adapted eGFR thresholds for CKD diagnosis are a subject of scientific debate, the 2024 KDIGO guidelines recommend the same approach for diagnosis in adults of all ages [2]. The global prevalence of CKD has been reported as approximately 13.6%, with the prevalence of CKD in stages 3–5 being 10.6% [3].

Changes in albuminuria are used as surrogate markers for the progression of kidney disease, as well as a surrogate endpoint to assess the efficacy of different treatments [4].

Malnutrition is a common feature in aging and chronic diseases. In patients with CKD, none of the available treatments can halt its progression, and the presence of malnutrition makes the situation even more problematic, being associated with prolonged hospitalization and high mortality [5]. The pathogenic mechanisms underlying malnutrition in CKD are complex and involve a combination of physiological changes, including gastrointestinal symptoms related to nephropathy, encephalopathy, pericarditis, loss of appetite, albumin depletion, reduced nutrient intake, ghrelin and leptin hormonal imbalances, metabolic disturbances, and gut dysbiosis [6]. The diagnosis of malnutrition is indicated by the presence of non-volitional weight loss, low body mass index (BMI), low muscle mass, reduced food intake or assimilation, and disease burden [7]. Even discrete changes associated with normal aging can increase the nutritional risk for older adults. Compared with younger adults, malnutrition in older individuals is both more common and may have a greater impact on outcomes, including physical function, healthcare utilization, and hospitalization [8,9]. Multiple nutritional screening and diagnostic tools have been proposed to identify patients at high risk of malnutrition, such as the Subjective Global Assessment (SGA) and the Malnutrition Inflammation Score, which have been validated for malnutrition screening in the context of CKD. However, the application of these questionnaire-based tools in elderly populations may be limited due to cognitive decline and communication-related problems [10]. For the elderly, the Mini Nutritional Assessment-Short Form (MNA) and the geriatric nutritional risk index (GNRI) have attracted considerable attention, as they provide simple but objective and comprehensive nutritional assessments [11]. The MNA is a validated nutrition screening and assessment tool addressed to patients aged 65 and above, originally comprised of 18 questions and now of 6 questions, that can identify individuals who are malnourished or at risk of malnutrition. The GNRI, which combines BMI and serum albumin levels to predict the risk of malnutrition, is another indicator designed to evaluate the nutritional status of geriatric individuals [8].

Anthropometric measurements and serum biomarkers are also indicative of malnutrition. Body composition can be assessed via computer tomography (CT) scanning, which can measure the visceral fat area from a single CT slice at L3 and can distinguish fat from tissues; however, its widespread use is limited by high costs, low accessibility, and radiation exposure. Bioelectrical impedance analysis (BIA) also allows for the evaluation of body composition, has the advantage of better access due to portability and affordability, and can measure fat and lean body mass [12].

Understanding the impact of various measures on outcomes is important, as the combination of malnutrition, kidney dysfunction, and senescence is associated with a higher risk of mortality. Advancements in the nutrition domain come as a response to the desire to prolong lifespans with quality and the overall well-being of every person. In elderly individuals and in those with CKD, the need to conserve autonomy as long as possible is a main driver of nutrition research. A growing body of literature has reported the specific benefits of nutritional interventions in CKD-associated malnutrition across the disease spectrum [13]. A malnutrition risk-based approach can guide the timing and implementation of nutritional interventions, matching this approach to predicted benefit. However, we still need more data regarding the magnitude of this problem, as well as identifying the most important factors that impact malnutrition in the context of elderly CKD.

The main purpose of this study was to identify the relationship between nutrition, kidney function, and albuminuria in a cohort of elderly patients using different assessment tools. The secondary objectives were to evaluate the prevalence of albuminuria, CKD, and malnutrition in our geriatric population.

## 2. Materials and Methods

The study included hospitalized patients aged 65 years and older who agreed to participate in all evaluations and follow the study protocol. The participants in our study were recruited from a geriatric clinic during routine health assessments. Patients with acute diseases, acute inflammation, dialysis, surgical procedures, active malignancy, terminal illness, or in palliative care programs were excluded.

A comprehensive assessment of nutritional status and a renal involvement evaluation of the patients was performed. Demographic data such as age, gender, smoking, and alcohol consumption habits were registered. The presence of comorbidities such as diabetes mellitus, arterial hypertension, and heart insufficiency was recorded. Anthropometric evaluation included body weight, height, body mass index (BMI), tricipital skinfold, and abdominal circumference.

The blood tests for nutrition status assessed serum albumin, total cholesterol, HDL- and LDL-cholesterol, triglycerides, and 25-OH vitamin D. We also measured the uric acid and C-reactive protein (CRP) levels.

The computed tomography (CT) scan measured the visceral fat area (VFA) from a single slice at the lumbar L3 level. The normal VFA ranges at about 10% of the total body fat. As expected, a parallel increase in VFA should be seen with an increase in total body fat. Siemens Healthineers (Forchheim, Germany). SOMATOM go Up was the CT scanner used for this evaluation.

Bioelectrical impedance analysis (BIA) was performed to measure total fat body mass and visceral fat. We used a validated device, the Visbody S30 Bioelectrical Impedance Body Composition Analyser Scanner (Visbody Intelligent Technology Co., Ltd., Xi’an, China).

The MNA short form consists of 6 questions about food intake, weight loss, BMI, mobility, and stress. It preserves the accuracy and validity of the original MNA in identifying elderly people who are malnourished or at risk of malnutrition. The MNA score ranges from 0 to 14, and patients scoring 0–7 points were considered malnourished. We considered 2 categories: patients having MNA score ≤ 7 indicated malnutrition, while the rest of the patients had MNA score > 7.

The GNRI score includes 4 grades of nutrition-related risk, defined as major risk (GNRI: <82), moderate risk (GNRI: 82 to <92), low risk (GNRI: 92 to ≤98), and no risk (GNRI: >98).

We assessed the renal function through the measurements of serum urea and creatinine. The glomerular filtration rate (eGFR) was estimated using the CKD-Epidemiology Collaboration (EPI) 2021 equation. Albuminuria was detected as the urinary albumin over creatinine ratio (UACR) from a urinary spot. Considering the grading of albuminuria, three categories of albuminuria were established: A1, which is normal or mildly elevated (UACR < 30 mg/g); A2, moderately elevated (UACR 30–300 mg/g); and A3, severely elevated (UACR > 300 mg/g).

### Statistics

Descriptive statistics were performed using box and whisker plots. Quantitative variables are described using the mean and standard deviation or median and percentiles, according to the normal distribution. Qualitative ordinal variables are described using the median and percentiles. Qualitative variables or ordinal variables with less than 7 categories are described with absolute and relative frequencies. As inferential statistics, variables were compared by subgroup using Student *t*-tests in the case of equal and unequal variances and using the Mann–Whitney test. Correlations were assessed with the Spearman correlation coefficient, as the variables of interest had multiple outliers. Multivariate linear regression was performed, with the dependent variables transformed into ranks and with the independent variables that were significant in the univariate analysis. As some variables had missing completely at random data (>6%), multivariate models were tested with and without these variables. The threshold value considered for statistical significance was 0.05. The analysis was performed using Microsoft Excel and SPSS version 25.0.

## 3. Results

The descriptive statistics for the entire cohort are presented in Table 1.

The study included 793 patients with an average age of 80 years, of which 29.6% were males and 4.9% were smokers. The score for median BMI was 27.75 kg/m^2^, that for abdominal circumference was 101 cm, that for eGFR was 69.04 mL/min/m^2^, and that for UACR was 20 mg/g. Median BIA body fat was 32.4%, and visceral fat was 11%. Using the cut-off of ≤7 for the MNA score, 274 patients had malnutrition, corresponding to 34.6% (Table 1).

According to the albuminuria levels, 478 patients (60.3%) had UACR < 30 mg/g, 260 patients (32.8%) had UACR 30–300 mg/g, and 55 patients (6.9%) had UACR > 300 mg/g.

The cohort was split into two groups. The first group included patients with CKD, defined as eGFR < 60 mL/min or UACR ≥ 30 mg/g for more than 3 months. The second group included patients with GFR ≥ 60 mL/min and UACR < 30 mg/g. The two groups were compared. In the group with CKD, patients were older, with fewer smokers and more females, lower HDL-cholesterol, higher triglycerides, and lower MNA scores (Table 2).

Correlation studies were performed with albuminuria and eGFR as independent variables with respect to the various dependent variables.

Increased albuminuria was correlated with increased age; low declared alcohol consumption; low body weight, BMI, and abdominal circumference; low serum albumin, vitamin D, and total and HDL-cholesterol; low adipose tissue and low visceral fat on bioimpedance assessment; and decreased renal function (high urea and creatinine and low eGFR) (Table 3).

A multivariate analysis including all factors presenting a significant correlation in the univariate analyses was performed. Because of missing completely at random data (>6%) in some variables, models with and without those variables were tested, and we reported the significant ones. This was the case for CRP, MNA score, BIA body fat, and BIA visceral fat. Higher albuminuria was determined by increased age (*p* = 0.010), low alcohol score (*p* = 0.032), and low HDL-cholesterol (*p* = 0.015).

We compared the nutritional markers according to the three categories of albuminuria levels.

There was a significant difference in age between the three subgroups of UACR (*p* < 0.001), but no significant differences in weight; BMI; abdominal circumference; total, HDL-, and LDL-cholesterol; and VFA were observed between these three subgroups.

**Figure 1:**
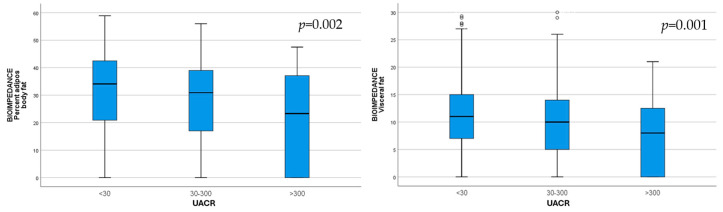
Body fat (*p* = 0.002) and visceral fat (*p* = 0.001) assessed via bioimpedance, decreased with increasing UACR category.

As the albuminuria category increased, BIA body fat (*p* = 0.002) and BIA visceral fat (*p* = 0.001) were significantly lower, indicating malnutrition (Figure 1).

**Figure 2:**
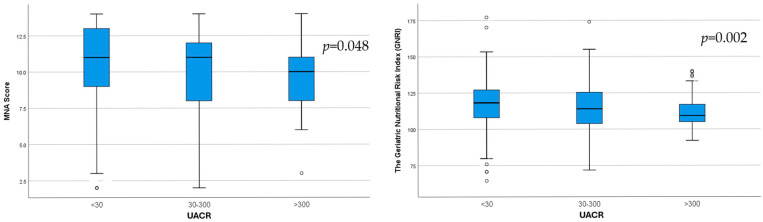
Differences in MNA score (*p* = 0.048) and GNRI score (*p* = 0.002) between the three UACR subgroups.

Increasing UACR category was associated with significantly lower MNA (*p* = 0.048) and GNRI (*p* = 0.002) scores (Figure 2).

The serum albumin levels (*p* < 0.001) were significantly lower, and the CRP levels (*p* < 0.001) were significantly higher with increasing UACR category.

The correlations between eGFR and variables for the entire cohort are presented in Table 4. Decreased eGFR was correlated with increased age, higher UACR, high uric acid, and the presence of malnutrition indicators (low serum albumin, low HDL-cholesterol, high triglycerides, and low MNA score) (Table 4).

A multivariate analysis including all factors presenting a significant correlation in the univariate analyses was performed. Lower eGFR was determined by increased age (*p* < 0.001), high triglycerides (*p* = 0.001), and high uric acid (*p* < 0.001).

There were 316 patients with eGFR < 60 mL/min/1.73 m^2^, corresponding to a prevalence of CKD G3a–5 of 39.84%. For this subgroup of CKD G3a–5 patients, the regression analysis determined correlations between different factors and renal function estimated through eGFR (Table 5). Decreased eGFR was correlated with increased UACR, high uric acid and CRP, and malnutrition indicators (low serum albumin; low total, LDL-, and HDL-cholesterol; high triglycerides; low MNA score; and low GNRI score); see Table 5.

For patients with an eGFR < 60 mL/min/1.73 m^2^, a multivariate analysis was performed to determine factors influencing the renal function estimated through eGFR, through which lower eGFR was determined by low albumin (*p* = 0.001), high triglycerides (*p* = 0.006), and high uric acid (*p* = 0.001).

**Figure 3:**
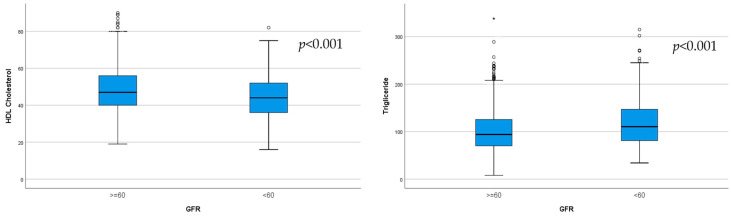
Significantly lower HDL-cholesterol (*p* < 0.001) and higher triglycerides (*p* < 0.001) were observed in the subgroup with eGFR < 60 mL/min/1.73 m^2^.

The HDL-cholesterol and triglyceride levels significantly differed between the two subgroups, determined according to eGFR (*p* < 0.001; Figure 3).

**Figure 4:**
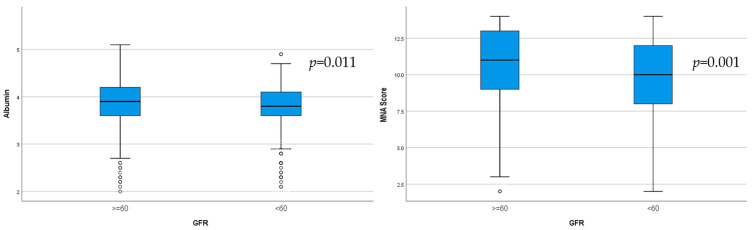
Serum albumin levels and MNA score in the two groups determined according to GFR (*p* = 0.001).

Significantly lower albumin (*p* = 0.011) and lower MNA score (*p* = 0.001), indicating malnutrition, were observed in the group of CKD patients (Figure 4).

There were no significant differences between CKD stages regarding the risk of malnutrition evaluated with respect to the GNRI score (*p* = 0.695).

## 4. Discussion

The first important finding of this study is the elevated prevalence of CKD in our geriatric patients. The demographic shift toward an older population worldwide has led to a notable increase in the prevalence of CKD, with global rates rising from 9.1% in 2017 to a significant 14.3% in 2023 [14]. Regarding the epidemiology of CKD, a recent review estimated the devastating impact of CKD, which is expected to become the fifth highest cause of years of life lost globally by 2040 [15]. A recent study from a neighboring geographical area reported prevalences of 25.03% and 34.6% in CKD stages 3a–5 and 1–5, respectively, in a geriatric ambulatory population [16]. The authors excluded patients with repeated hospitalizations. Our study focused on hospitalized elderly patients having more severe health problems (including kidney disease) than elderly outpatients, which explains the higher observed prevalence of CKD stages 3a–5 (39.84%). Nevertheless, an alternative hypothesis is that the fixed thresholds proposed in the KDIGO guidelines could potentially lead to overdiagnosis and overestimation of the prevalence of CKD among elderly populations [2,17]. Similar results on the prevalence of CKD (44%) in individuals aged ≥ 65 years have been reported for Kidney Early Evaluation Program (KEEP) and National Health and Nutrition Examination Survey (NHANES) participants [18]. A study from China reported a prevalence of 87% regarding malnutrition in CKD patients [12].

The second finding of our study is the high prevalence of malnutrition. According to the MNA scores, 34.6% of patients were malnourished. The MNA, which has high sensitivity and reproducibility [11], showed a good relationship with outcomes and proved to be an adequate tool to describe nutritional risk in elderly CKD patients. As additional advantages, the MNA is easy to interpret, requires only a short time for application, and is well accepted by the elderly. MNA can even be used to screen elderly patients in acute medical situations in general wards for malnutrition or risk of malnutrition [19]. As measured using the MNA, malnutrition or risk of malnutrition ranges from 40.1% (reported in older residents of nursing homes in Spain) to 46% (in tertiary hospitals in China) [19]. The MNA streamlines the screening process for malnutrition. Early detection of this risk paves the way for an early nutritional approach and preventing undesirable outcomes with respect to the health of older individuals [20,21].

The third important finding of this study is that increased albuminuria was significantly correlated with most of the measured malnutrition-related indices (low body weight, BMI and abdominal circumference, low serum albumin, vitamin D, total and HDL-cholesterol, low adipose tissue and low visceral fat on BIA, and low MNA and GNRI scores), increased age, and lower renal function (high urea and creatinine and low eGFR) in the enrolled patients. We identified notable correlations between decreased eGFR and low serum albumin, low HDL-cholesterol, high triglycerides, and low MNA score, emphasizing that malnutrition was also significantly associated with more advanced kidney disease in the enrolled patients. Albuminuria acts as an early indicator of kidney damage and a significant predictor of the progression of CKD [22]. The close association between albuminuria and low eGFR highlights its significance as a public health issue, emphasizing the importance of early clinical screening and intervention, especially in at-risk populations [23]. The prevalence of moderately elevated albuminuria is reported to be between 5 and 19% in the general population, with rates as high as 23% observed in hypertensive patients and 40% in diabetic patients. In our study, moderately elevated albuminuria was present in 32.8% of patients, and 39.7% of patients had a UACR > 30 mg/g. The observed correlation between age and albuminuria may reflect age-related renal changes, but this association should be interpreted with caution in the absence of detailed comorbidity data.

A connection between obesity and urinary loss of albumin through hyperfiltration as a pathogenic mechanism recognized in focal segmental glomerulosclerosis points to a positive relationship between fat accumulation and albuminuria. Various obesity-related indices (BMI, waist-to-hip ratio, visceral adiposity index, body adiposity index) have been significantly associated with albuminuria in patients with diabetes mellitus [24]. A cross-sectional study that enrolled over 40,000 participants aged 40 or older from China demonstrated an association between a body shape index and an elevated urinary albumin–creatinine ratio in Chinese community adults [25]. Conventional epidemiological approaches in a UK Biobank study suggested that higher waist-to-hip ratio and BMI are independently and positively correlated with albuminuria, indicating an association between central obesity and albuminuria [26]. Munkhaugen et al. [27] evaluated 75,000 volunteers in a cohort study conducted over a 20-year period in Norway and found a strong association between BMI and the risk of kidney disease.

In light of previous studies, while our results seem surprising, the geriatric population is a particular category and, furthermore, it should be noted that the relationship between malnutrition and kidney disease is variable. The inverse correlation of albuminuria with low fat indicators and the correlation between eGFR decline and malnutrition in our study may be explained by the reverse epidemiology described in patients with CKD. This concept is the consequence of significant associations, indicating that BMI is an inverse predictor of mortality in CKD patients [28]. The BMI paradox has been described in CKD, with particular impacts of obesity, inflammation, and atherosclerosis on outcomes [29]. An inverse association between lipid levels and mortality has been reported in men with CKD who are not yet on dialysis, signaling once more that malnutrition has a paradoxical impact on mortality in the CKD case as opposed to the general population [30]. Malnutrition, identified through the use of SGA and serum albumin, has been correlated with the results of a qualitative examination of urinary protein in a study in China on 426 CKD patients [12]. A lack of association between epicardial adipose tissue thickness and adverse cardiovascular events in patients undergoing hemodialysis has even been reported, highlighting the need for further exploration of the relationships between the kidneys and nutritional status [31]. No relationships could be demonstrated between BMI, obesity, and the prevalence of CKD in a recent meta-analysis analyzing the global prevalence of CKD [3]. In our study, the risk of malnutrition, identified in terms of a low GNRI score, was correlated with albuminuria and decreased eGFR in the subgroup with eGFR < 60 mL/min/1.73 m^2^. In a model adjusted for age and sex in a CKD-ROUTE trial, patients with lower GNRI values had 4.09 times greater risk of CKD compared with those with higher GNRI values. The secondary analysis of the CKD-ROUTE study reported that the GNRI is associated with all-cause mortality in older patients with CKD. One of the conclusions of this trial is that malnutrition appears to be an effective predictor of clinical outcomes in patients with early-stage CKD, suggesting that the optimal timing for nutritional assessment and intervention may be during early-stage CKD [32].

However, the relationship between malnutrition and albuminuria has not been extensively studied, which limits our current understanding of potential risk factors for malnourished CKD in geriatric populations. Malnutrition in the elderly is multifactorial, often due to undernutrition, reduced appetite, or chronic illness. Similarly, albuminuria is likely to reflect underlying comorbidities such as diabetes or vascular disease. It is important to avoid overinterpretation, as observed associations do not imply direct causality. Future studies should examine the associations of other measures of adiposity with outcomes in CKD. Debates regarding whether a missing factor influences the obesity paradox, such as proteinuria, are ongoing [33]. Malnutrition is a crucial condition among older inpatients, both as a cause and consequence of disease [34]. We can observe that the reverse epidemiology of malnutrition and obesity with adverse outcomes shifts from hemodialysis populations to earlier stages of CKD [35,36]. We can only hypothesize that advanced age may have an influence on this relationship. We found a difference in age between the groups—those with GFR < 60 mL/min and an albumin/creatinine ratio <30 mg/g were older than those with GFR > 60 mL/min and a ratio ≥30 mg/g. This is expected given the natural progression of kidney disease with age, and it is also possible that malnutrition may be partly attributed to age. However, age was statistically controlled for in the multivariate analysis, and the same results were observed after adjustment.

The last important finding of this study is the impact of inflammation on albuminuria and decreased eGFR. Even if CRP was correlated with eGFR, it did not remain statistically significant in the multivariate analysis, meaning that CRP was not an independent predictor of eGFR. The role of inflammation in health and CKD is very important, and previous studies have reported links with the Malnutrition Inflammation Score. A newer inflammation index has recently been shown to be significantly associated with eGFR and all-cause mortality in CKD patients [37,38]. Malnutrition can have multiple causes, and chronic low-grade inflammation, as reflected by elevated CRP, may worsen nutritional status. In our study, we did not specifically examine the impact of elevated CRP on malnutrition, so we cannot draw direct conclusions in this regard. It is possible that some of our patients with higher inflammatory markers had poorer nutritional indicators, but this hypothesis requires further investigation. Future studies should specifically assess the relationship between inflammation (including CRP) and malnutrition in this population.

There are several limitations to this study. First, data on some important variables that can influence CKD and albuminuria—such as the history of comorbidities and CKD etiology—were lacking. Second, the study included elderly patients with CKD, regardless of its duration, and the duration of CKD can influence the effects of the disease on malnutrition. Third, the serum creatinine-based eGFR may overestimate renal function in sarcopenic or undernourished patients, so future studies should include cystatin-based eGFR also. Fourth, although active malignancy was an exclusion criterion, we acknowledge that subclinical or undiagnosed neoplasms might have been present, and cancer-related glomerulopathies or cancer-related cachexia may have contributed to the observed findings in some patients. Finally, this study was cross-sectional in design, so causal relationships and long-term clinical outcomes could not be confirmed. Nonetheless, the results may help to shed light on the relationships between malnutrition-related indicators, albuminuria, and CKD in elderly populations. Further prospective studies are needed to evaluate the development and progression of malnutrition in patients with CKD.

Although it has some limitations, our study is noteworthy, as it was carried out in a large cohort of elderly people and was able to identify an important number of patients with CKD and malnutrition. In addition, our study analyzed several modalities to evaluate malnutrition and highlighted that poor nutrition (reflected by low albumin and cholesterol, low anthropometric measures, low adiposity on BIA, and low MNA and GNRI scores) correlated with albuminuria. Our study also suggests a dual relationship between inflammation and albuminuria. At the same time, low eGFR was also correlated with some of the abovementioned malnutrition indicators in elderly patients. The relationship between malnutrition and albuminuria has been relatively less defined in geriatric CKD patients, and most research has highlighted albuminuria associated with obesity. Finally, although the study was cross-sectional, the large sample and high significance of the results enhance the value of our findings.

## 5. Conclusions

In conclusion, in a geriatric population, elevated albuminuria was found to be correlated with malnutrition. Even though albuminuria is recognized as a marker of renal health, it was correlated with more adverse complications—such as malnutrition—in our study. Therefore, personalized nutritional evaluation plans should be integrated into the comprehensive care of elderly CKD patients, even to mildly increase albuminuria levels, with the definitive purpose of preventing and treating malnutrition effectively.

## Figures and Tables

**Table 1:** Characteristics of the patients in the study cohort.

	All (793 Patients)
Age	80 (75; 85)
Male, no (%)	235 (29.6)
Diabetes, no (%)	298 (37.6)
Hypertension, no (%)	736 (92.8)
Smoking, no (%)	39 (4.9)
Alcohol	0 (0; 0)
Weight (kg)	71 (60; 82.5)
BMI (kg/m^2^)	27.75 (23.53; 31.93)
Arm skinfold	20 (12; 29)
Abdominal circumference	101 (91; 111)
Albumin (g/dL)	4.2 (3.9; 4.5)
Total cholesterol (mg/dL)	171 (142; 202)
HDL-cholesterol (mg/dL)	46 (38; 55)
LDL-cholesterol (mg/dL)	100.1 (76.4; 130.2)
I confirmTriglycerides (mg/dL)	99 (75; 135)
CRP (mg/dL)	0.51 (0.19; 1.98)
Urea (mg/dL)	47 (39; 63)
Creatinine (mg/dL)	0.91 (0.74; 1.19)
eGFR (mL/min/m^2^)	69.04 (46.97; 86.36)
UACR (mg/g)	20.75 (10.91; 58.39)
Uric acid (mg/dL)	6 (4.9; 7.2)
Vitamin D (ng/mL)	22.42 (13.82; 32.71)
BIA body fat	32.4 (19.4; 41)
BIA visceral fat	11 (6; 14)
VFA	176.41 (103.07; 253.65)
GNRI	116.37 ± 15.53
GNRI ≤ 98, no (%)	78 (11)
MNA Score	11 (8; 12)
MNA ≤ 7, no (%)	274 (34.6)

Data are expressed as median (25th–75th percentile) or numerical value (percentages). eGFR, estimated glomerular filtration rate; UACR, urinary albumin over creatinine ratio; BIA, bioimpedance assessment; VFA, visceral fat area; GNRI, Geriatric Nutritional Risk Index; MNA, Mini Nutritional Assessment; CRP, C-reactive protein.

**Table 2:** Comparison between groups.

	GFR ≥ 60 and UACR < 30 (311 Patients)	GFR < 60 or UACR ≥ 30 (482 Patients)	*p*
Age	77 (71; 82.5)	82 (76; 87)	**<0.001**
Male, no (%)	105 (33.8)	130 (27)	**0.041**
Diabetes, no (%)	101 (32.6)	197 (41)	**0.017**
Hypertension, no (%)	282 (90.7)	454 (94.6)	**0.035**
Smoking, no (%)	25 (8)	14 (3)	**0.001**
Alcohol	0 (0; 0)	0 (0; 0)	0.70
Weight (kg)	72.7 (61.35; 84.45)	70.2 (59; 82)	0.226
BMI (kg/m^2^)	27.9 (23.85; 31.92)	27.68 (23.41; 31.95)	0.929
Arm skinfold	20 (14; 30)	20 (12; 28)	0.128
Abdominal circumference	101 (92; 111)	101 (90.5; 111)	0.795
Albumin (g/dL)	4.3 (4; 4.6)	4.1 (3.8; 4.4)	**<0.001**
Total cholesterol	173 (146.4; 204)	168.5 (137; 201)	0.164
HDL-cholesterol	49 (41; 57)	44 (36; 53)	**<0.001**
LDL-cholesterol	99.2 (80.2; 130.8)	100.9 (72.9; 127.7)	0.792
Triglycerides	94 (73.5; 123)	105 (76; 141)	0.06
CRP (mg/dL)	0.34 (0.16; 0.85)	0.66 (0.25; 2.44)	**<0.001**
Urea	41 (34; 50)	54 (43; 75)	**<0.001**
Creatinine (mg/dL)	0.77 (0.69; 0.88)	1.08 (0.85; 1.38)	**<0.001**
eGFR (mL/min/m^2^)	84.5 (72.98; 93.35)	51.5 (37.51; 75.06)	**<0.001**
UACR (mg/g)	11.89 (8.57; 17.27)	42.97 (16.48; 108.99)	**<0.001**
Uric acid (mg/dL)	5.4 (4.3; 6.5)	6.5 (5.2; 7.8)	**<0.001**
Vitamin D (ng/mL)	24.68 (15.73; 33.57)	20.89 (12.6; 32.38)	**0.029**
BIA body fat	32.25 (20.5; 42.7)	32.4 (18.4; 40.3)	0.245
BIA visceral fat	11 (7; 14)	11 (6; 14)	0.071
VFA	157.53 (93.81; 235.94)	185.18 (107.01; 263.78)	0.468
GNRI	117.47 ± 15.2	115.58 ± 15.74	0.110
MNA Score	11 (9; 13)	10 (8; 12)	**0.002**
MNA malnutrition, no (%)	103 (42.4)	171 (52.3)	**0.019**

Data are expressed as median (25th–75th percentile) or numerical value (percentages). eGFR, estimated glomerular filtration rate; UACR, urinary albumin over creatinine ratio; BIA, bioimpedance assessment; VFA, visceral fat area; GNRI, Geriatric Nutritional Risk Index; MNA, Mini Nutritional Assessment; CRP, C-reactive protein. Bold characters are used to highlight statistical significance.

**Table 3:** Correlations between albuminuria and study variables.

	UACR, mg/g (n = 793)		
	Spearman Correlation Coefficient	*p*	95% Confidence Interval
Age	0.246	**<0.001**	(0.18; 0.31)
Alcohol Score	−0.112	**0.002**	(−0.18; −0.04)
Weight (kg)	−0.146	**<0.001**	(−0.21; −0.08)
BMI (kg/m^2^)	−0.126	**0.001**	(−0.2; −0.06)
Arm skinfold	−0.071	0.053	(−0.2; −0.07)
Abdominal circumference	−0.120	**0.001**	(−0.19; −0.05)
Albumin	−0.222	**<0.001**	(−0.29; −0.15)
Total cholesterol	−0.073	**0.040**	(−0.14; 0)
HDL-cholesterol	−0.098	**0.006**	(−0.17; −0.03)
LDL-cholesterol	−0.048	0.179	(−0.12; 0.02)
Triglycerides	−0.009	0.795	(−0.08; 0.06)
CRP	0.235	**<0.001**	(0.15; 0.32)
Urea	0.144	**<0.001**	(0.07; 0.21)
Creatinine	0.086	**0.015**	(0.02; 0.16)
eGFR (mL/min/m^2^)	−0.132	**<0.001**	(−0.2; −0.06)
Uric acid	0.065	0.073	(−0.01; 0.13)
Vitamin D	−0.102	**0.004**	(−0.17; −0.03)
BIA body fat	−0.171	**<0.001**	(−0.25; −0.09)
BIA visceral fat	−0.165	**<0.001**	(−0.24; −0.09)
VFA	−0.039	0.566	(−0.17; 0.09)
GNRI Score	−0.192	**<0.001**	(−0.26; −0.12)
MNA Score	−0.157	**<0.001**	(−0.24; −0.08)

eGFR, estimated glomerular filtration rate; UACR, urinary albumin over creatinine ratio; BIA, bioimpedance assessment; VFA, visceral fat area; GNRI, Geriatric Nutritional Risk Index; MNA, Mini Nutritional Assessment; CRP, C-reactive protein. Bold characters are used to highlight statistical significance.

**Table 4:** Correlations between eGFR and study variables.

	eGFR, mL/min/m^2^ (793 patients)		
	Spearman Correlation Coefficient	*p*	95% Confidence Interval
UACR	−0.132	**<0.001**	(−0.2; −0.06)
Age	−0.306	**<0.001**	(−0.37; −0.24)
Alcohol Score	0.044	0.215	(−0.03; 0.11)
Weight (kg)	0.033	0.361	(−0.04; 0.1)
BMI (kg/m^2^)	0.004	0.921	(−0.07; 0.07)
Arm skinfold	0.060	0.099	(−0.12; 0.02)
Abdominal circumference	−0.001	0.979	(−0.07; 0.07)
Albumin	0.085	**0.017**	(0.01; 0.15)
Total cholesterol	0.043	0.228	(−0.03; 0.11)
HDL-cholesterol	0.182	**<0.001**	(0.11; 0.25)
LDL-cholesterol	0.046	0.199	(−0.02; 0.12)
Triglycerides	−0.216	**<0.001**	(−0.28; −0.15)
CRP	−0.153	**0.001**	(−0.24; −0.06)
Uric acid	−0.536	**<0.001**	(−0.58; −0.48)
Vitamin D	0.053	0.142	(−0.02; 0.12)
BIA body fat	−0.007	0.857	(−0.08; 0.07)
BIA visceral fat	0.005	0.895	(−0.07; 0.08)
VFA	−0.118	0.085	(−0.25; 0.02)
GNRI Score	0.048	0.198	(−0.03; 0.12)
MNA Score	0.162	**<0.001**	(0.08; 0.24)

eGFR, estimated glomerular filtration rate; UACR, urinary albumin over creatinine ratio; BIA, bioimpedance assessment; VFA, visceral fat area; GNRI, Geriatric Nutritional Risk Index; MNA, Mini Nutritional Assessment; CRP, C-reactive protein. Bold characters are used to highlight statistical significance.

**Table 5:** Correlations between eGFR and variables in the subgroup with eGFR < 60 mL/min/1.73 m^2^.

	eGFR, mL/min/1.73 m^2^		
	Spearman Correlation Coefficient	*p*	95% Confidence Interval
UACR	−0.126	**0.026**	(−0.23; −0.02)
Age	−0.059	0.296	(−0.17; 0.05)
Alcohol score	−0.054	0.337	(−0.16; 0.06)
Weight	−0.039	0.498	(−0.15; 0.07)
BMI	0.013	0.822	(−0.1; 0.13)
Arm skinfold	0.032	0.586	(−0.16; 0.06)
Abdominal circumference	−0.013	0.827	(−0.13; 0.1)
VFA	−0.172	0.108	(−0.37; 0.04)
MNA score	0.158	**0.023**	(0.02; 0.29)
Albumin	0.268	**<0.001**	(0.16; 0.37)
Total cholesterol	0.130	**0.021**	(0.02; 0.24)
HDL-cholesterol	0.244	**<0.001**	(0.14; 0.35)
LDL-cholesterol	0.116	**0.042**	(0; 0.22)
Triglycerides	−0.157	**0.005**	(−0.26; −0.05)
Uric acid	−0.379	**<0.001**	(−0.47; −0.28)
Vitamin D	0.092	0.103	(−0.02; 0.2)
BIA body fat	0.065	0.305	(−0.06; 0.19)
BIA visceral fat	0.046	0.466	(−0.08; 0.17)
GNRI score	0.140	**0.021**	(0.02; 0.26)
CRP	−0.147	**0.039**	(−0.28; −0.01)

eGFR, estimated glomerular filtration rate; UACR, urinary albumin over creatinine ratio; BIA, bioimpedance assessment; VFA, visceral fat area; GNRI, Geriatric Nutritional Risk Index; MNA, Mini Nutritional Assessment; CRP, C-reactive protein. Bold characters are used to highlight statistical significance.

## Data Availability

The research data supporting this study’s findings are not publicly available. Further inquiries can be directed to the corresponding authors. The dataset used during the current study is available from the corresponding authors upon reasonable request.
